# Contribution of cardiac surgeons in transcatheter aortic valve replacement activity in France

**DOI:** 10.1093/icvts/ivaf068

**Published:** 2025-03-14

**Authors:** Gabriel Saiydoun, Saadé Saadé, Chadi Aludaat, Chloé Bernard, Guillaume Lebreton, Thomas Modine, Thierry Folliguet, Charles Juvin, Maroua Eid, Xavier-Benoit D’Journo, Louis Labrousse, Michel Kindo, Julien Guihaire, Jean-Marc Baste, Olivier Fouquet, Marylou Para, Johann Cattan, Mohammed Alghamdi, Guillaume Guimbretiere, Olivier Bouchot, André Vincentelli, Pascal Leprince, Jean-Philippe Verhoye

**Affiliations:** Department of Cardiac Surgery, Pitié Salpetrière University Hospital, Sorbonne University, Paris, France; Department of Cardiac Surgery, Henri Mondor University Hospital, UPEC, Creteil, France; Department of Cardiac Surgery, Strasbourg University Hospital, Nouvel Hôpital Civil, Strasbourg, France; Department of Thoracic and Cardiac Surgery, Rouen University Hospital, Rouen, France; Department of Cardiac Surgery, Dijon University Hospital, Dijon, France; Department of Cardiac Surgery, Pitié Salpetrière University Hospital, Sorbonne University, Paris, France; Department of Cardiac Surgery, Bordeaux University Hospital, Bordeaux, France; Department of Cardiac Surgery, Henri Mondor University Hospital, UPEC, Creteil, France; Department of Cardiac Surgery, Pitié Salpetrière University Hospital, Sorbonne University, Paris, France; Department of Cardiac Surgery, Angers University Hospital, Angers, France; Department of Thoracic Surgery, Marseille University Hospital, Marseille, France; Department of Cardiac Surgery, Bordeaux University Hospital, Bordeaux, France; Department of Cardiac Surgery, Strasbourg University Hospital, Nouvel Hôpital Civil, Strasbourg, France; Department of Cardiac Surgery, Marie-Lannelongue Hospital, Le Plessis-Robinson, France; Department of Thoracic and Cardiac Surgery, Rouen University Hospital, Rouen, France; Department of Cardiac Surgery, Angers University Hospital, Angers, France; Department of Cardiac Surgery, Bichat University Hospital, Paris, France; Department of Cardiac Surgery, Bordeaux University Hospital, Bordeaux, France; Department of Cardiac Surgery, Pitié Salpetrière University Hospital, Sorbonne University, Paris, France; Department of Cardiothoracic Surgery, Nantes University Hospital, Nantes, France; Department of Cardiac Surgery, Dijon University Hospital, Dijon, France; Department of Cardiac Surgery, Lille University Hospital, Lille, France; Department of Cardiac Surgery, Pitié Salpetrière University Hospital, Sorbonne University, Paris, France; Department of Cardiac Surgery, Rennes University Hospital, Rennes, France

**Keywords:** TAVR, cardiac surgery, Heart Team, aortic stenosis

## Abstract

**OBJECTIVES:**

This study provides a thorough analysis of cardiac surgeons’ involvement in transcatheter aortic valve replacement (TAVR) activities in France, covering decision-making, procedural roles, training and outcome analysis.

**METHODS:**

A nationwide survey was sent to all cardiac surgeons and all cardiac surgery trainees in France. Subgroup analysis was performed for age, status (established versus in-training) and type of practice facility.

**RESULTS:**

A total of 172 surgeons from both private and public sectors responded to the survey. Most respondents (71%) had TAVR activity, and there were no significant differences between subgroups. Most respondents with TAVR activities (30%) had average access (once per week). Almost one-third of centres had >3 established surgeons with TAVR activity, whereas 19% had no in-training surgeons with TAVR activity. TAVR was the only structural practice for 67% of surgeons, while 33% practiced other structural procedures. When asked, 82% of surgeons were against establishing TAVR programmes in centres without a cardiac surgery programme. Most TAVR patients (72%) were discussed by the Heart Team, and only 9% of surgeons said their relationship with the interventional cardiologist was disrupted. Two-thirds of vascular complications were managed by cardiac surgery, and only 6% of cardiac surgeons admitted were unfit to handle any vascular complications.

**CONCLUSIONS:**

In France, cardiac surgeons are becoming increasingly involved in TAVR procedures as an integral part of the Heart Team.

## GRAPHICAL ABSTRACT

**Figure:**
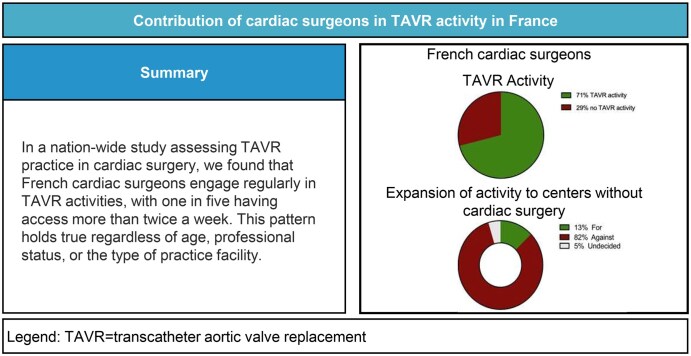

## INTRODUCTION

Aortic stenosis (AS) is a deteriorating disease that lacks effective medical treatment and poses a bleak prognosis if left unmanaged [1–3]. For individuals with severe AS, transcatheter aortic valve replacement (TAVR) demonstrates good feasibility in patients deemed inoperable or at intermediate risk and has emerged as a successful alternative to surgical aortic valve replacement (SAVR) [4–6]. In recent years, the use of TAVR has surpassed that of SAVR in numerous countries. The reasons for this shift are the lower risk and reduced recovery time [7, 8]. While the prevalence of AS remains generally stable at 0.5–1% of the general population in developed countries [9, 10], the pool of patients eligible for TAVR has expanded annually, creating a continual demand for establishing new centres.

In 2020 and 2024, the French High Health Authority (*HAS-Haute autorité de santé*) issued a report to determine eligibility criteria for TAVR. To guarantee patient safety, only centres with a cardiac surgery programme within the same building and a volume of over 200 SAVRs per year were authorized to perform TAVR. The report also stipulated that centres should have multidisciplinary Heart Teams that include cardiac surgeons, interventional and clinical cardiologists and a cardiac anaesthesiologists [11]. Given the significant increase in TAVR procedures due to expanded indications and widespread adoption, a new report is planned to reassess the criteria for overseeing TAVR implantation centres by 2025. This will involve refining the criteria for recommending TAVR procedures and their execution in the operating room, both in terms of technical setup and team composition, as well as considering the possibility of offering these procedures at centres without on-site cardiac surgery. TAVR procedures in France are primarily performed in cardiac surgery centres within university hospitals (CHU) in major cities. Additionally, some general hospitals (CH) in cities near these urban centres also offer TAVR, ensuring broader regional accessibility. Beyond public institutions, private centres with cardiac surgery activity also perform TAVR, typically located near CHUs, facilitating collaboration and rapid patient transfer if needed. This distribution ensures that TAVR remains available in specialized, high-volume centres while maintaining strict patient safety standards. However, rural hospitals generally do not perform TAVR, as access to emergency cardiac surgery remains a key requirement.

This study aimed to provide a nationwide survey of the involvement and participation of cardiac surgeons in TAVR activities in France, including decision-making, procedural roles, training and outcome analysis.

## MATERIALS AND METHODS

### Ethical statement

This study was approved by the institutional review board (IRB00012919) of the French Society of Thoracic and Cardio-Vascular Surgery (CERC-SFCTCV-2024–05-28_34824).

### Electronic survey

An electronic survey was sent via email to all cardiac surgeons and cardiac surgery trainees listed in the French Society of Thoracic and Cardiovascular Surgery (SFCTCV) database. Anonymity was required for all participants. The survey was successfully delivered to 216 surgeons and trainees on the 11th of April 2023. Reminders were sent on the 11th of May and on the 30th of June 2023. The survey was sent to 216 cardiac surgeons and fellow trainees involved in structural heart procedures, out of a total of 474 professionals practicing cardiac surgery in France. A total of 172 participants from all French regions completed the survey with response rate 79.6%.

### Statistical analysis

Subgroup analysis was performed for age, status and type of practice facility. Young surgeons were divided into two subgroups: 24–35 and 36–45 years old. Senior surgeons were divided into two subgroups: 46–55 and 56–67 years old. First-year residents, residents, research year residents, fellows and junior doctors were considered in training, while hospital practitioners, academic hospital practitioners with a faculty position and private practice hospital practitioners were considered established surgeons. Hospital practitioner (PH—Praticien Hospitalier) is a senior surgeon with a permanent hospital-based position in the French healthcare system. They have full autonomy in their clinical practice and perform surgeries. Private practice and mixed practice centres were considered private practice centres, while all other centres were classified as public sector institutions.

The response rate was calculated as the proportion of completed responses among all invited participants. To estimate the precision of this proportion, the margin of error (MoE) at a 95% confidence level was calculated using the standard formula for proportions:


MoE = Z × √[(p(1 - p))/n]

where for a 95% confidence level, is the observed response rate (172/216 = 0.796), and is the total number of invited participants (216). Applying this formula, the MoE was calculated to be 5.3%, meaning that the true response rate is expected to lie within this range in 95% of cases.

Statistical analyses were performed using Prism (Prism 5, GraphPad Software, San Diego, CA).

## RESULTS

### Population characteristics

The majority of respondents (86%) were male, 98% practiced adult cardiac surgery, and 66% worked in a public university hospital (Table 1). Among the survey respondents, the 24–35 age group was the most represented with 33% of respondents, while the 56–67 age group was the least represented with 19% of respondents. Paris was the most represented city, with 14% of participants declaring practicing there. Private practitioners were the most common status, representing 25% of respondents, and first-year residents represented only 2% of all respondents.

**Table 1:** Population characteristics

Sex (*n* = 172)	
Male	86%
Female	14%
Age group (*n* = 172)	
24-35 yo	33%
36-45 yo	28%
46-55 yo	20%
56-67 yo	19%
City (*n* = 172)	
Paris	14%
Bordeaux	11%
Marseille	6%
Lyon	6%
Other	62%
Nice	1%
Status (*n* = 172)	
First-year resident	2%
Resident	12%
Resident (research year)	2%
Junior doctor	5%
Fellow	18%
Public hospital practitioner	18%
Academic hospital practitioner	19%
Private practice hospital practitioner	25%
Activity (*n* = 172)	
Adult	98%
Congenital	2%
Centre (*n* = 172)	
University hospital	66%
Private hospital	22%
Private non-profit health institution	7%
Non university public hospital	3%
Mixt	2%

yo: years old.

### TAVR practice

**Figure 1:**
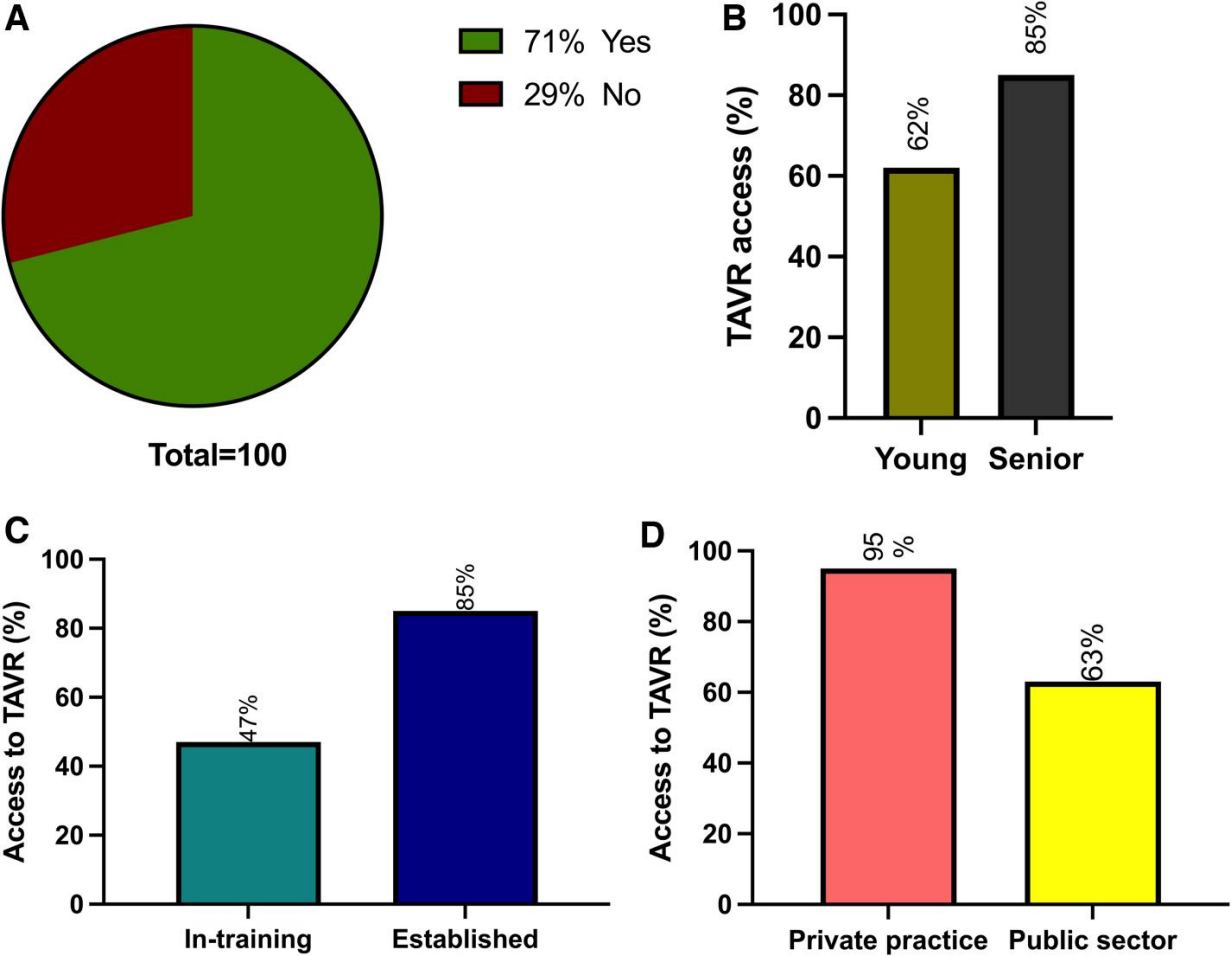
TAVR practice among cardiac surgeons. (**A**) TAVR activity: yes (green) and no (red). (**B**) TAVR access in young (khaki green) and senior (dark grey) surgeons. (**C**) TAVR access in in-training (teal) or established (dark blue) surgeons. (**D**) TAVR access in private practice (salmon) and public sector (yellow)

**Figure 2:**
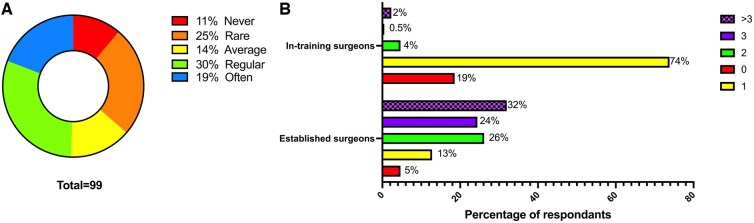
(**A**) TAVR activity frequency (never: 0/week, red; rare: 1/month, orange; average: 1/week, yellow; regular: 3/month, green; often: >2/week, blue). (**B**) Number of cardiac surgeons with a TAVR activity in established and in-training surgeons (none: red, one: yellow, two: green, three: solid purple, more than three: patterned purple)

**Figure 3:**
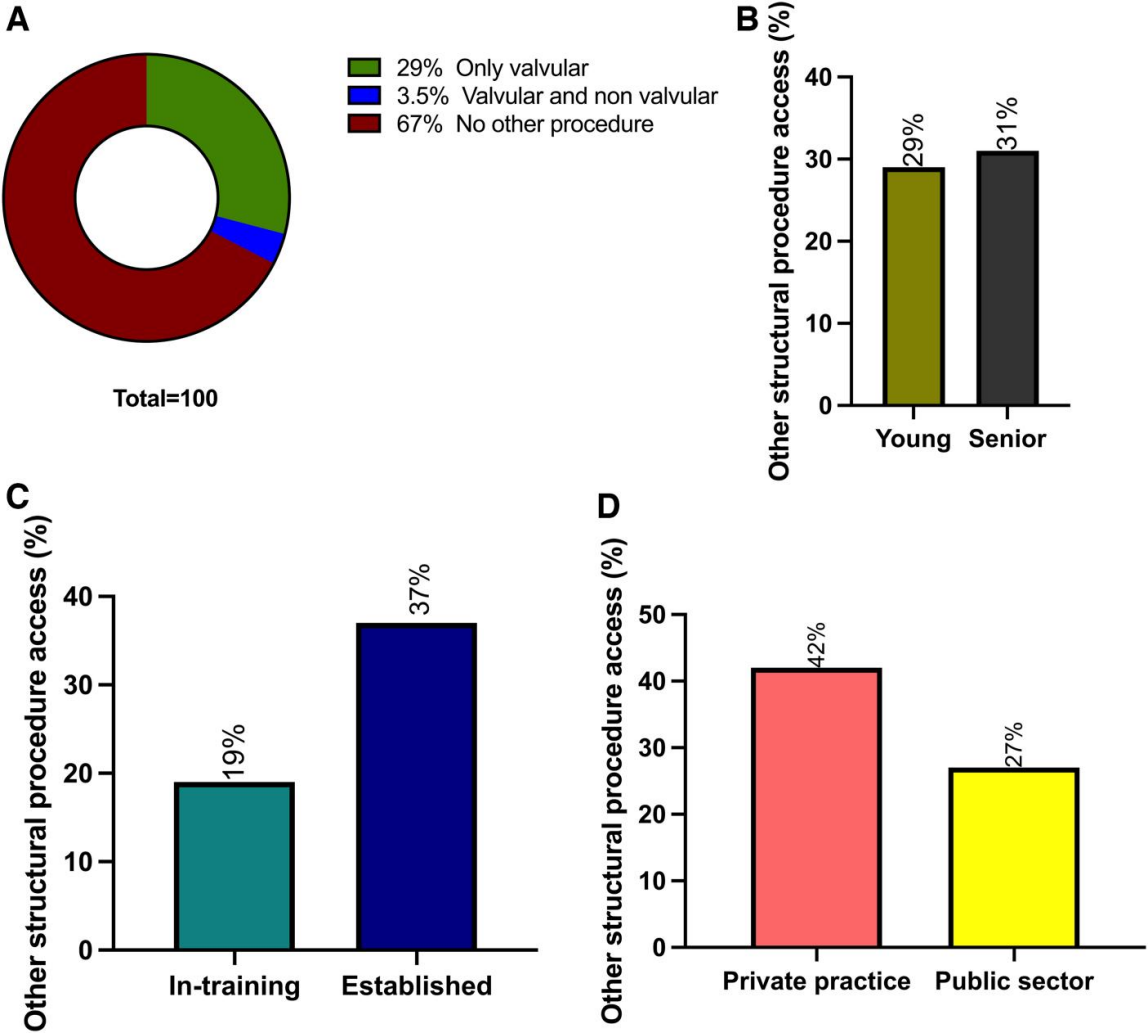
Other structural and interventional activities practiced by cardiac surgeons. (**A**) Distribution of practices: no other procedure (burgundy), only valvular interventional procedures (green), valvular and non-valvular interventional procedures (blue). (**B**) Other structural and interventional activities in young (khaki) and senior (dark grey) surgeons. (**C**) Other structural and interventional activities in in-training (teal) or established (dark blue) surgeons. (**D**) Other structural and interventional activities in private practice (salmon) and public sector (yellow)

More than two-thirds (71%) of surgeons performed TAVR activity (Fig. 1A). More senior and established surgeons had access to TAVR than their younger counterparts (Fig. 1B and C). Most surgeons practicing in private practice had access to TAVR, compared to only two-thirds of surgeons practicing in public practice (Fig. 1D). Among the survey respondents, the most common TAVR access frequency among cardiac surgeons was once per week (30%). Additionally, 19% had frequent access to TAVR (more than twice per week), 14% had regular access (three times per month), 25% had rare access (once per month) and 11% did not have access to TAVR procedures (Fig. 2A). Concerning in-training surgeons, 74% of centres reported having one in-training surgeon practicing TAVR, while 19% reported none, and a minority of centres (2%) reported >3 (Fig. 2B). As for established surgeons, 32% of centres had >3 established surgeons practicing TAVR, and only 5% reported none. Most cardiac surgeons (67%) did not have structural activity other than TAVR, while 29% performed other valvular interventional procedures and 3.5% performed other valvular and non-valvular interventional procedures (Fig. 3A). Among those who underwent other interventional procedures, there were no significant differences between age (Fig. 3B), status (Fig. 3C) or type of practice facility (Fig. 3D) subgroups.

### TAVR activity expansion

**Figure 4:**
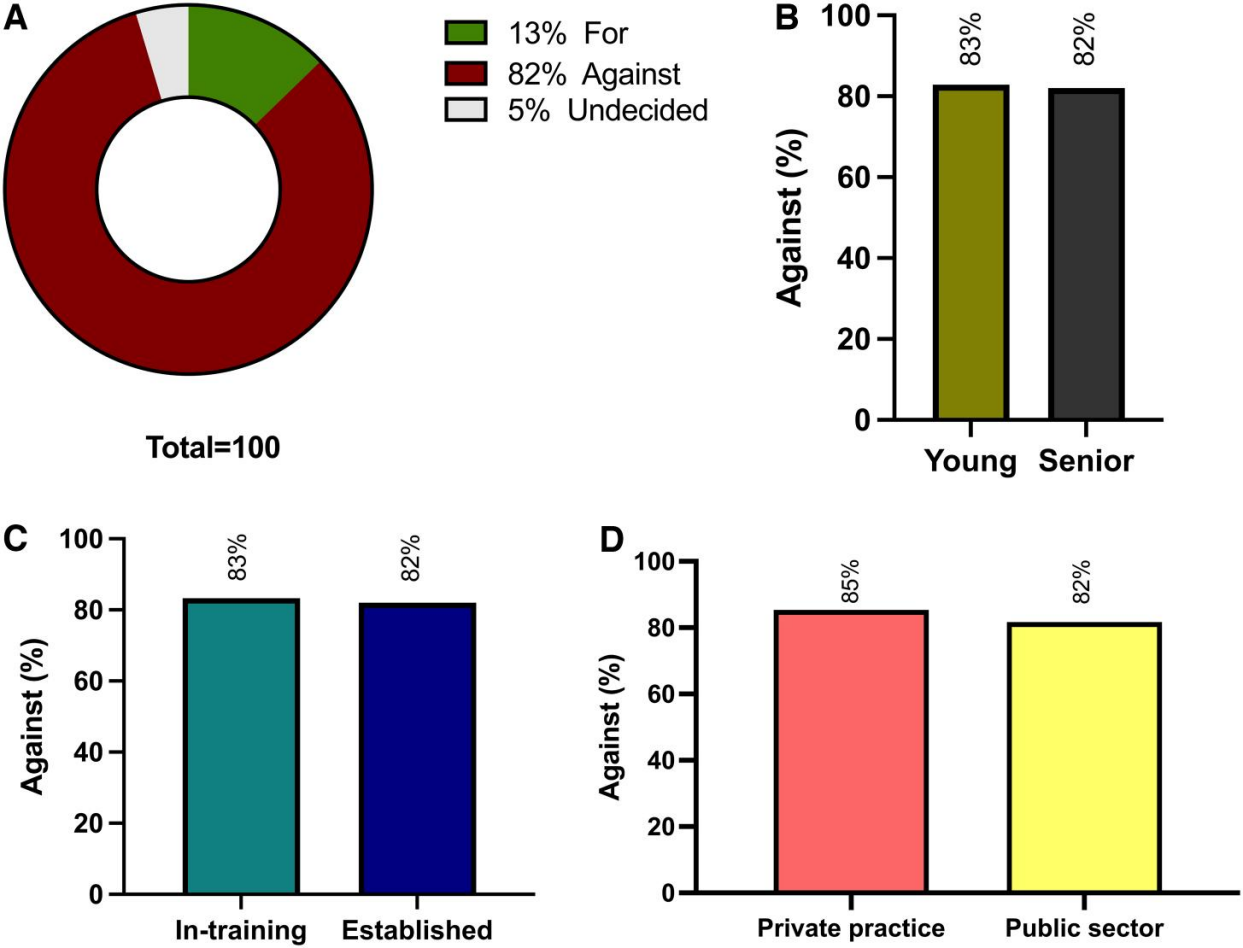
TAVR Expansion. (**A**) Opinion of cardiac surgeons; against (burgundy), for (green), undecided (grey). (**B**) TAVR expansion opinion in young (khaki) and senior (dark grey) surgeons. (**C**) TAVR expansion opinion in in-training (teal) or established (dark blue) surgeons. (**D**) TAVR expansion opinion in private practice (salmon) and public sector (yellow)

Precisely, 82% of cardiac surgeons were against expanding TAVR activity to centres without on-site cardiac surgery programmes, while 13% were in favour, and 5% were undecided (Fig. 4A). There were no significant differences between age (Fig. 4B), status (Fig. 4C) or type of practice facility (Fig. 4D) subgroups.

### Heart Team

**Figure 5:**
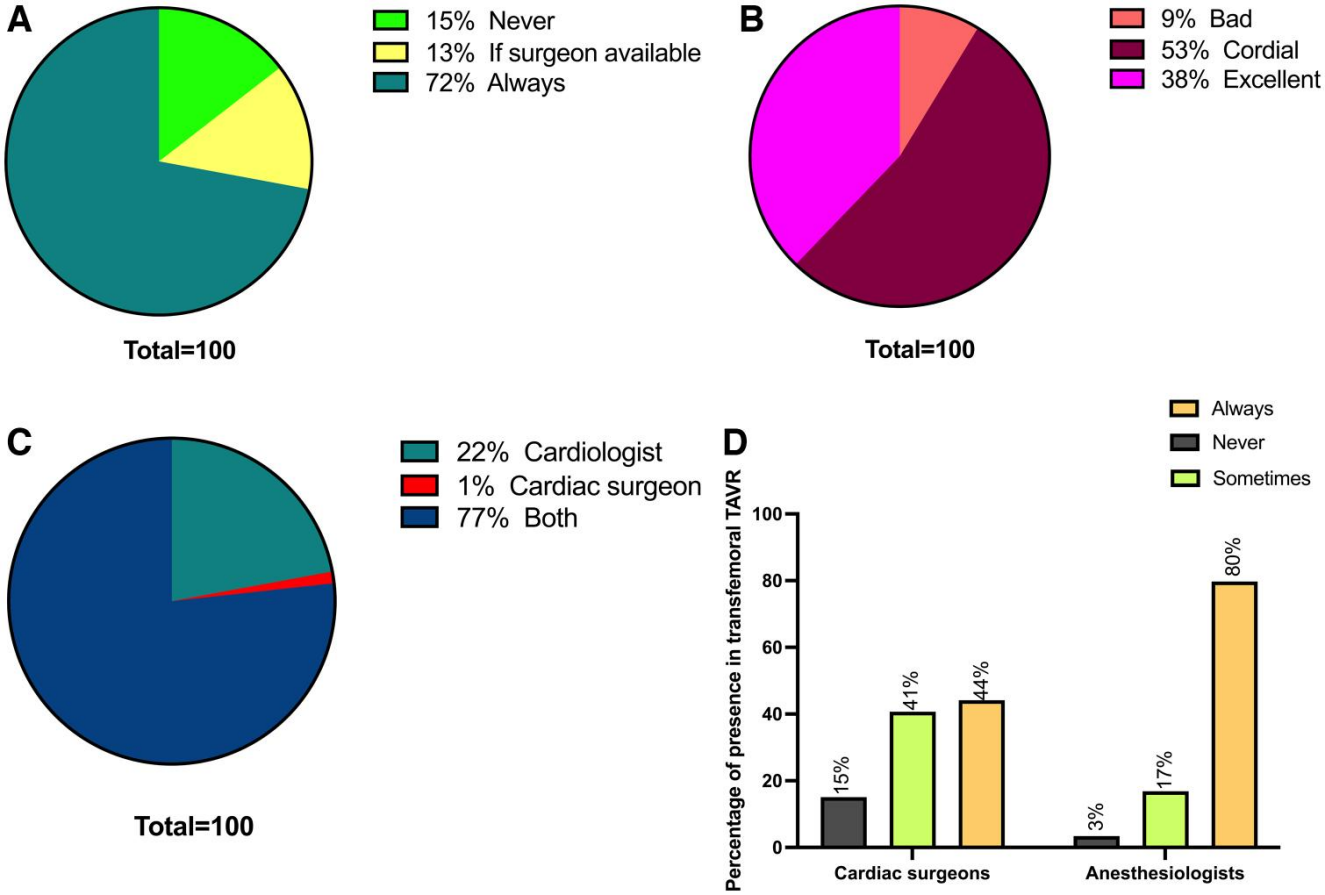
The Heart Team. (**A**) Percentage of TAVR patients discussed in the Heart Team: always (teal), if surgeon is available (yellow), and never (green). (**B**) Relationship with interventional cardiologist: excellent (pink), cordial (burgundy) and bad (orange). (**C**) TAVR patient’s recruitment is done by: cardiologist (teal), cardiac surgeon (red) and both (dark blue). (**D**) Percentage of presence of cardiac surgeon and cardiac anaesthesiologist in transfemoral TAVR: never (dark grey), sometimes (light green) and always (yellow)

Discussing TAVR indications in a Heart Team setting was reported by 72% of respondents, while 15% said that there was no referral of TAVR patients to the Heart Team (Fig. 5A). Only 9% reported a strained relationship with the interventional cardiology team, but most had a cordial or excellent relationships (Fig. 5B). Both the interventional cardiologist and cardiac surgeons recruited TAVR patients in 77% of cases and only by the cardiac surgeon in only 1% of cases (Fig. 5C). In transfemoral TAVR cases, 44% reported the permanent presence of a cardiac surgeon and 15% reported that the cardiac surgeon was not present (Fig. 5D). Eighty percent of transfemoral TAVR cases were mainly performed in the presence of an anaesthesiologist/certified-registered nurse anaesthetist.

### Procedural complications

**Figure 6:**
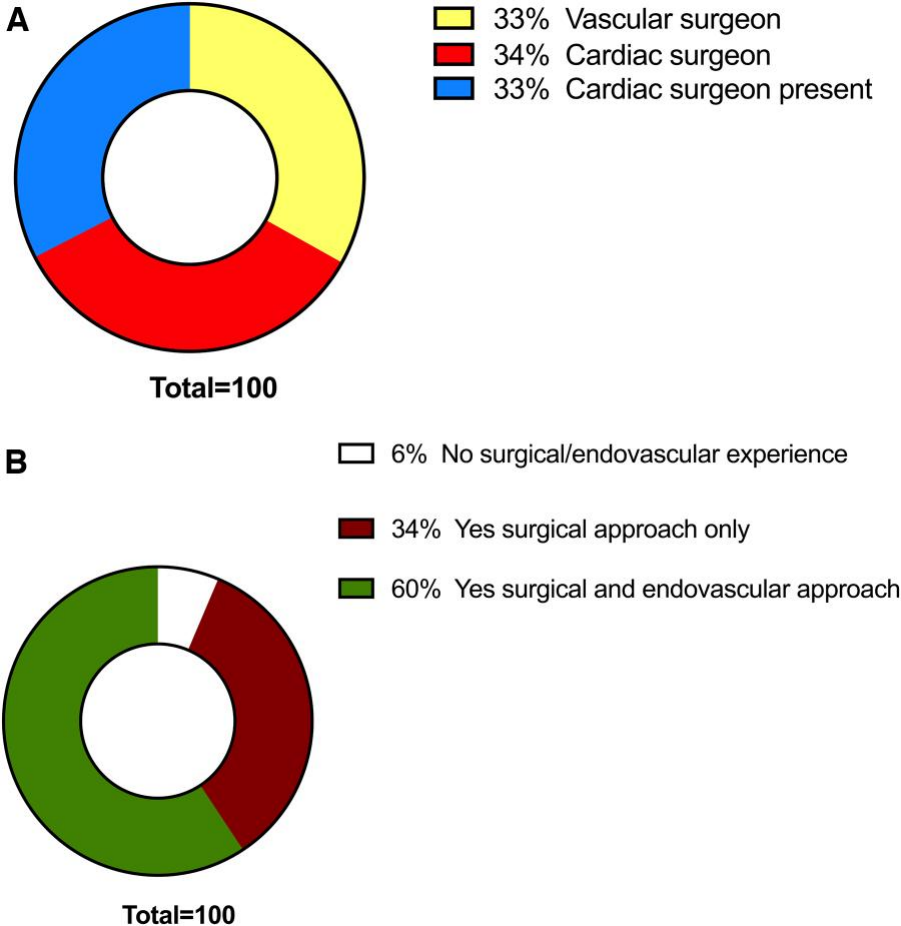
Procedural complications. (**A**) In case of vascular complications, who is called: vascular surgeon (yellow), cardiac surgeon (red) and cardiac surgeon always present (blue). (**B**) Ability to manage vascular complications: surgical and endovascular approaches (green), surgical approach only (burgundy) and neither (white)

In the event of a vascular complication, 33% called the vascular surgeon, 34% the cardiac surgeon, and in 33% of cases, the cardiac surgeon was always present to be consulted if needed (Fig. 6A). Sixty percent cardiac surgeons reported being able to manage a vascular complication both by endovascular and open approaches (Fig. 6B).

### TAVR training and ability

Regarding TAVR training, 53% relied on the TAVR industry-sponsored training programmes, 13% on a specific official diploma, 16% on residency lectures and 74% said autonomous learning contributed to their TAVR skills (Supplementary Material). Among cardiac surgeons, 49% reported being fully able to properly select TAVR patients, 42% to correctly anticipate and treat medical complications, 64% to correctly anticipate and treat surgical complications, 40% to correctly size aortic annulus and choose a TAVR valve, 56% to be fully able to perform an endovascular approach to complications, 68% to perform an open approach, 47% to master interventional skills and 42% to master TAVR-specific skills. These skills entail to contextualize the statistic.

## DISCUSSION

This study provides insights into the current scenario of cardiac surgeons participating in TAVR activities in France. These findings are both fascinating and informative, revealing that cardiac surgeons exhibit a high level of engagement and active involvement throughout all stages of the TAVR procedure. Our results align with those from the Society of Thoracic Surgeons (STS) database, albeit with a slight difference in timeframe [12]. The STS data indicated that 77.5% of cardiac surgeons were involved in TAVR activity at their institution, while our study showed that 71% of cardiac surgeons had TAVR activity. On the other hand, surgeons in the USA exhibited higher TAVR volume, with most surgeons (40.5%) performing five to nine cases per month, whereas numbers were much lower in France. In line with North American registries, our research demonstrated that French cardiac surgeons play a crucial role as essential members of the Heart Team. This underscores their significance, countering emerging studies that propose a diminishing role for surgeons [13–15].

TAVR has evolved swiftly since open-heart operations ceased to be the exclusive option for AS. Originally designated for the elderly and those in critical health, TAVR is now commonly performed and has been extended to patients with an intermediate risk. Recent studies have shown that the risk of conversion to open heart surgery from TAVR is <1% [7, 16, 17]. However, these studies also underscored the potential for mortality rates of up to 50% in the event of severe complications, such as annular rupture. Should TAVR activity be expanded to centres without cardiac surgery, mortality rates are expected to rise, as the severity of these complications, albeit rare, could have dire consequences. This can also be partially attributed to the inverse correlation between higher procedural volumes and better TAVR outcomes [18, 19]. Although there is a continuously increasing demand from a growing number of patients eligible for TAVR, with mortality on waiting lists being non-negligible, the emphasis should be on extending TAVR activities to restructured centres equipped with adequate technical facilities. To avoid any disruption of equipoise, this TAVR expansion should include all members of the Heart Team, prioritizing procedural volume and reliable quality measurements, such as 30-day and 1-year TAVR mortality. This approach is especially appropriate because most French cardiac surgeons surveyed are against establishing TAVR activity at institutions without on-site cardiac surgery.

Moreover, contrary to popular beliefs, our study found that cardiac surgeons enjoy a good relationship with their counterparts in interventional cardiology. This underscores the importance that decisions regarding AS should not be influenced by specialty bias but rather guided by the appropriate use of evidence. In the context of coronary artery disease, the choice between coronary artery bypass grafting and percutaneous coronary intervention is influenced by whether the head of cardiology or cardiac surgery is present at the Heart Team meetings [20]. An ideal TAVR team combines essential and complementary skills, including patient selection, mastery of TAVR-specific techniques and complication management. Cardiac surgeons typically handle vascular complications through open and endovascular approaches, while interventional cardiologists manage coronary complications. An anesthesiologist specialized in cardiac surgery is crucial, yet 20% of TAVR procedures proceed without one. Lessons from vascular surgery underscore the need for specialized guidewire skills, but 18.59% of centres lack TAVR-trained residents. Standardized training across high-volume centres is essential, with official certification programmes for both surgery and cardiology residents, as in France. Proper training enables cardiac surgeons to independently perform transfemoral TAVR, achieving results comparable to major trials [21].

Undoubtedly, in the future, each cardiac surgeon must possess proficiency in TAVR, share a collective skill set with interventional cardiologists and engage in collaborative decision-making. It is now more crucial than ever for surgeons to apply their knowledge and experience in treating valvular disease, prompting a necessary shift in mindset across generations, supported by leadership and robust training programmes. The evolving landscape of TAVR underscores the necessity for cardiac surgeons to acquire proficiency in catheter-based interventions. Effective training is essential to meet this need, and studies show that structured programmes allow trainees to safely acquire the skills required for minimally invasive procedures like aortic valve replacement. This finding underscores the importance of structured training programmes in equipping surgeons with the essential skills required for TAVR procedures [22]. Additionally, a systematic review in the same journal emphasized the role of simulation-based training in cardiac surgery. The review concluded that such training is essential for developing the competencies necessary for minimally invasive techniques, including TAVR [23]. Other innovative technics like virtual reality platforms can enhance the visualization of patient-specific anatomy, thereby improving the accuracy of TAVR planning [24]. These studies collectively advocate for comprehensive training and a collaborative approach between cardiac surgeons and interventional cardiologists to ensure optimal patient outcomes in TAVR procedures.

The contemporary landscape of cardiovascular healthcare emphasizes the necessity for surgeons to proficiently utilize their expertise in treating valvular diseases, notably through procedures such as TAVR. This evolving medical frontier demands a generational mindset shift that is strongly supported by decisive leadership and extensive training programmes. TAVR surgeons must master a wide range of foundational skills that are crucial for the success of the procedure. These skills include precise femoral echo-guided puncture for vascular access, which is essential for safety and efficacy of the intervention. Managing large-bore closure devices is crucial for safeguarding the arterial entry point, both before and after the procedure, thereby preventing complications.

Mastering techniques for retrograde aortic valve crossing and managing temporary cardiac pacing during prosthesis deployment or valvuloplasty should be part of the expertise of the cardiac surgeon. Beyond these core skills, TAVR surgeons must also excel in advanced competencies, such as detailed cardiac CT assessments for meticulous surgical planning, vascular surgical hemostasis to manage bleeding, capacity to adjust to atypical circumstances through utilization of alternative arterial entry sites and the readiness to immediately convert to open surgery if complications arise. These combined proficiencies empower TAVR surgeons to adeptly navigate intricate clinical scenarios, thereby substantially improving patient outcomes and reaffirming their indispensable role in contemporary cardiovascular therapies.

These insights align with the findings of Clermidy *et al.* (2024) [25], who highlighted the challenges and opportunities in robotic-assisted thoracic surgery training in France, emphasizing the necessity for better access to practical training across institutions. This reflects a concerted effort within the surgical community to refine educational methodologies and prepare surgeons for the intricacies of modern cardiovascular interventions. Additionally, Hussein *et al.* (2022) [26] highlighted the necessity of objective assessment tools in cardiothoracic surgery training, noting that while validated methods exist, their integration into curricula remains limited. Their systematic review underscores the importance of structured evaluation in improving trainee progression, ensuring skill acquisition and enhancing transparency in surgical education.

### Limitations

This study has several limitations. First, it was an electronically only survey and only two reminders were sent. It included responses from 172 surgeons, representing 79.6% of the invited participants, ensuring a broad representation of the population of cardiac surgeons involved in TAVR activities in France. While no formal power calculation was conducted, this response rate suggests that the findings are reflective of real-world practices. However, subgroup comparisons should be interpreted with caution, as certain groups (e.g. in-training surgeons) may have a lower number of respondents, limiting statistical power. Future studies with larger, prospectively powered cohorts may help refine these findings further. Second, the small sample size represents a limitation, particularly for subgroup comparisons, as it may reduce statistical power and the ability to detect significant differences. Some subgroups contain a limited number of respondents, which may lead to variability in results.

## CONCLUSION

In summary, as an integral part of the cardiac team, cardiac surgeons are highly involved in the TAVR activity in France and are against its expansion to secondary or primary care facilities. Encouraging shared decision-making and fostering collaborative procedural execution allows each team member to contribute their experience and expertise to patient care. It is imperative that all stakeholders recognize the urgency of this matter as a critical issue in our specialty. This study calls for action from health system leadership, recommending specific policy changes, training enhancements and resource allocation.

## SUPPLEMENTARY MATERIAL

Supplementary material is available at *ICVTS* online.

## FUNDING

This work was supported by the French Society of Thoracic and Cardiovascular Surgery (SFCTCV) and the Association des Jeunes Chirurgiens Thoracique et Cardiovasculaire (AJCTCV).

## CONFLICT OF INTEREST

C.A. reports receiving training and educational support from Edwards Lifesciences, Medtronic and Abbott. T.M. is a consultant for Medtronic, Edwards Lifesciences and Abbott Structural. There are no other potential conflicts of interest to declare.

## Supplementary Material

ivaf068_Supplementary_Data

## Data Availability

Data are available after formal request to and acceptance by the Ethical Committee of Clinical Research of the French Society of Thoracic and Cardiovascular Surgery (comite-ethique@sfctcv.org).

## Notes

Presented at the Annual Meeting of the European Association for Cardio-Thoracic Surgery, Lisbon, October, 2024.

## ABBREVIATIONS

AS: Aortic stenosis
CRNA: Certified-registered nurse anaesthetists
SAVR: Surgical aortic valve replacement
STS: Society of Thoracic Surgeons
TAVR: Transcatheter aortic valve replacement
