# Supplementary material for: Contribution of cardiac surgeons in transcatheter aortic valve replacement activity in France
Source: Interdiscip Cardiovasc Thorac Surg. 2025 Mar 14;40(3):ivaf068. doi: 10.1093/icvts/ivaf068 (PMC11938354; doi:10.1093/icvts/ivaf068)
Supplement: ivaf068_Supplementary_Data [file ivaf068_supplementary_data.zip › CORRECT SUPPLEMENTARY MATERIAL.docx]

**SUPPLEMENTARY MATERIAL**

**Table S1 :** French Society of thoracic and cardiovascular surgery’s survey

| What is your age group?   - 24-35 years old - 36-45 years old - 46-55 years old - 56-67 years old |
| --- |
| What is your affiliated city?   - *City choice* |
| What is your profession?   - Adult cardiac surgery - Congenital cardiac surgery |
| What is your status?   - First year resident - Other resident - Resident (research year) - Junior doctor - Fellow - Public Hospital practitioner - Private Hospital practitioner - Academic Hospital practitioner |
| What type of center do you work in?   - University-hospital - Public hospital (non-university) - Private practice - Mixed activity center |
| What is your opinion on the regulated opening of new TAVI centres without cardiac surgery?   - For - Against - Undecided |
| Personally, do you practice TAVI procedures?   - Yes - No |
| The number of TAVI procedures in your center is between:   - 0-100 - 100-200 - 200-300 - >300 |
| How often do you have access to TAVI procedures?   - Never: 0/week, - Rare: 1/month - Average: 1/week - Regular: 3/month - Often: >2/week |
| Regarding TAVI activity, how many cardiac surgeons are directly involved in your center?   - 0 - 1 - 2 - 3 - >3 |
| How many in-training surgeons (residents, assistants, fellows) are involved or in TAVI training in your center?   - 0 - 1 - 2 - 3 - >3 |
| Personally, do you practice other structural procedures?   - No - Only valvular procedures - Valvular and non-valvular procedures |
| Would you like to receive training in TAVI?   - Yes, I am interested in having a regular TAVR activity. - Yes, although I don’t think I will have a regular TAVR activity. - No, I don’t think I will have a permanent position allowing a TAVR activity - No, I am not interested - I am already trained |
| Who provides your training?   - TAVR industrials - Specific diploma - Residency courses - Autonomous training - Conferences |
| TAVI procedures take place in:   - Hybrid setting - Operating theatre - Cath-lab - Many sites |
| If the procedure takes place in a hybrid room, it is located:   - Operating theatre - Interventional Cardiology - Radiology - There is more than one hybrid room - The procedure is not realized in a hybrid room |
| During a transfemoral percutaneous TAVI procedure, is a cardiac surgeon present in the room?   - Always - If surgeon’s available - Never |
| During a transfemoral TAVI procedure, is an anesthesiologist and/or cardiac surgery CRNA present?   - Always - Sometimes - Never |
| If you are involved in TAVI procedures, is there a Heart Team meeting with a cardiac surgeon present?   - Always - If surgeon’s available - Never |
| Patient files are discussed during the medical-surgical meeting:   - Always - Sometimes - Never |
| What is your relationship with the interventional cardiology team performing TAVI?   - Cordial - Excellent - Bad |
| Recruitment of eligible patients for TAVI is done:   - By the cardiologist - By the cardiac surgeon - Both |
| If a vascular complication occurs and no cardiac surgeon is in the room, the team in charge of the patient requests:   - Vascular surgeon - Cardiac surgeon - Cardiac surgeon always present |
| If a vascular complication occurs, are you able to manage this complication alone?   - Yes, surgical approach - Yes, surgical and endovascular approach - No |
| What is your level of expertise in selecting the right patient for TAVI?   - I don’t know how to do - I am able with help - I am able with occasional help - I am fully able |
| What is your level of expertise in anticipating, detecting, and managing medical complications (conduction disorders, renal insufficiency, stroke...)?   - I don’t know how to do - I am able with help - I am able with occasional help - I am fully able |
| What is your level of expertise in anticipating, detecting, and managing surgical complications (vascular, tamponade...)?   - I don’t know how to do - I am able with help - I am able with occasional help - I am fully able |
| What is your level of expertise in analyzing the CT scan, sizing, and selecting the valve to be implanted?   - I don’t know how to do - I am able with help - I am able with occasional help - I am fully able |
| What is your level of expertise in the management of percutaneous vascular access (ultrasound-guided puncture, pre-closing...)?   - I don’t know how to do - I am able with help - I am able with occasional help - I am fully able |
| What is your level of expertise in the management of surgical vascular access (carotid or axillary, excluding apical)?   - I don’t know how to do - I am able with help - I am able with occasional help - I am fully able |
| What is your level of expertise in basic interventional cardiology procedures (management of guides, probes, angiography)?   - I don’t know how to do - I am able with help - I am able with occasional help - I am fully able |
| What is your level of expertise in specific TAVI procedures (crossing, valve mounting, deployment...)?   - I don’t know how to do - I am able with help - I am able with occasional help - I am fully able |

CRNA, certified-registered nurse anaesthetists; CT, computed tomography; TAVR, transcatheter aortic valve replacement.

**
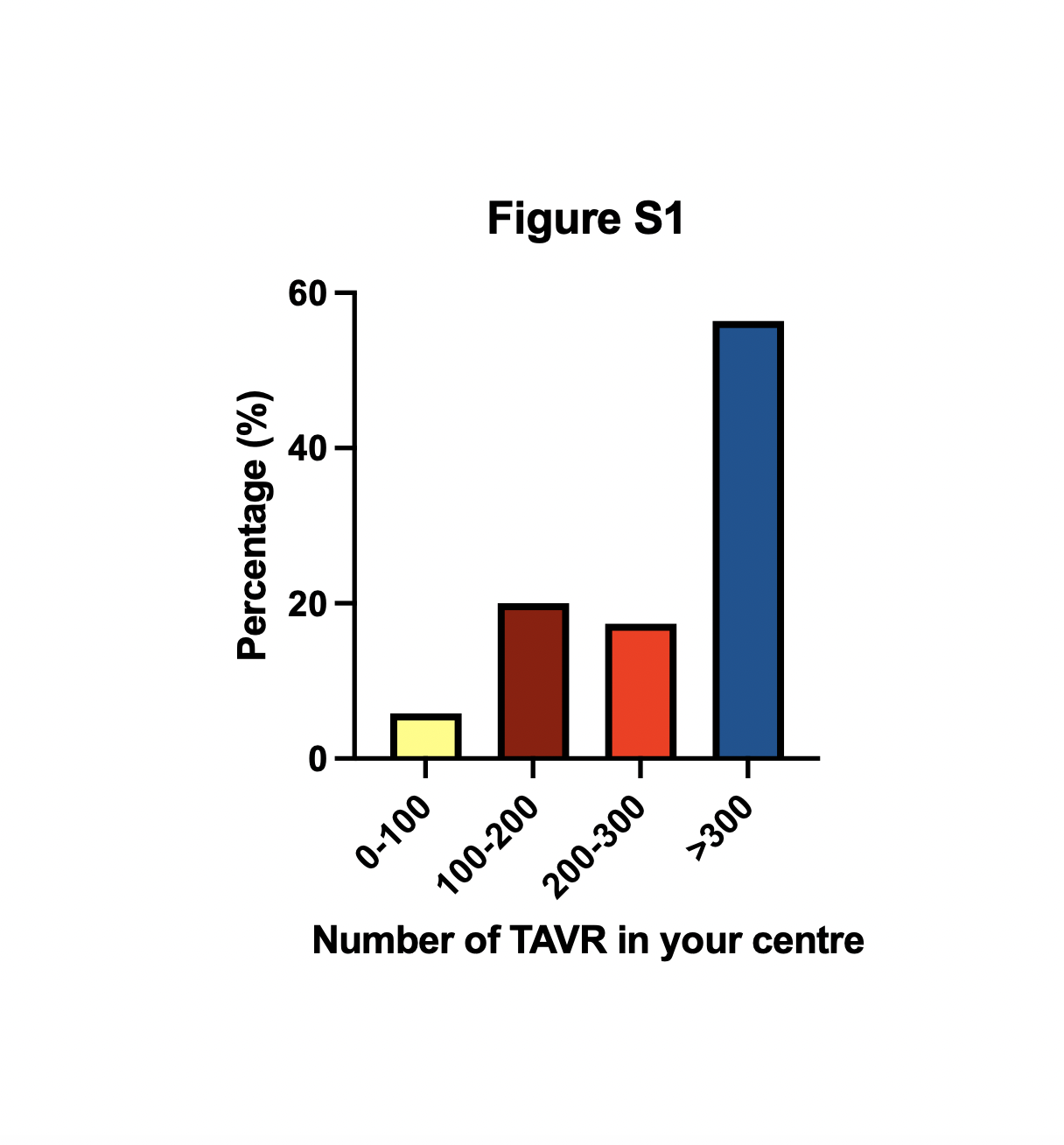
**

**Figure S1:** Number of TAVR In your centre: 0-100 (yellow), 100-200 (burgundy), 200-300 (red), >300 (blue).

**
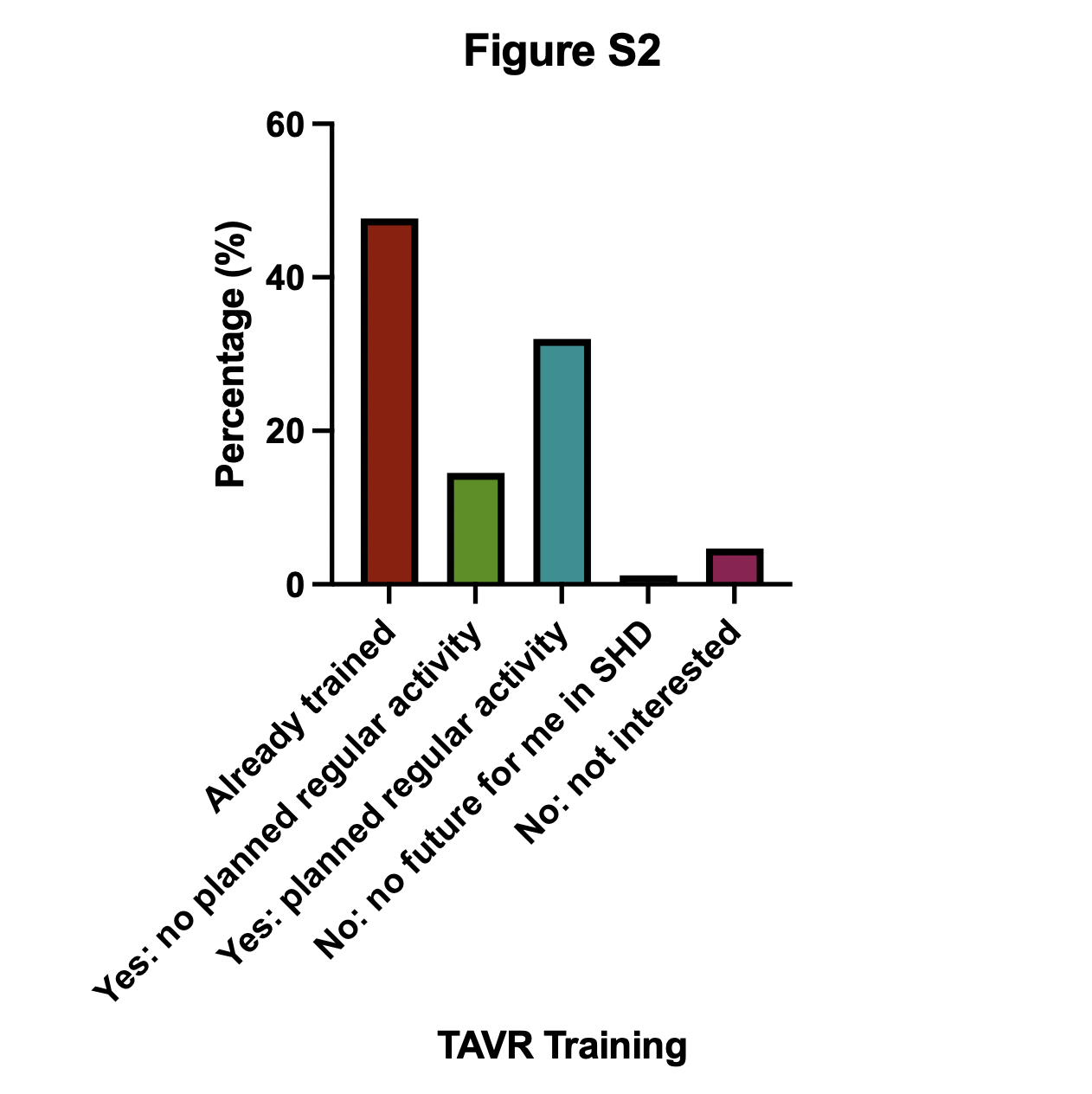
**

**Figure S2:** TAVR Training: already trained (burgundy), Yes: no planned regular activity (green), Yes: planned regular activity (teal), No: no future in SHD (black), No: not interested (purple).

**
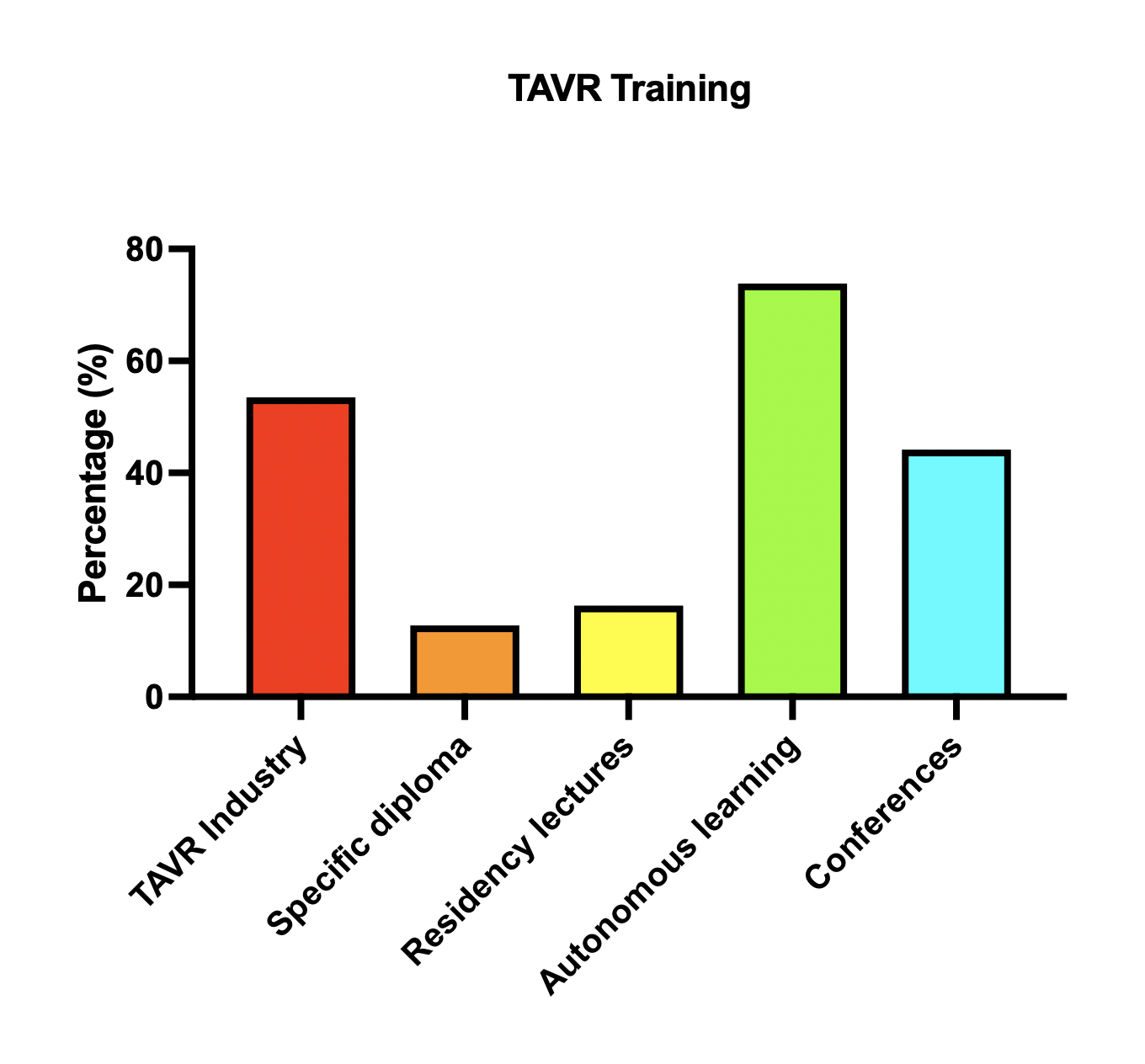
**

**Figure S3:** Who trains you? TAVR industry (red), specific diploma (orange), residency lectures (yellow), autonomous learning (green), conferences (blue).

**
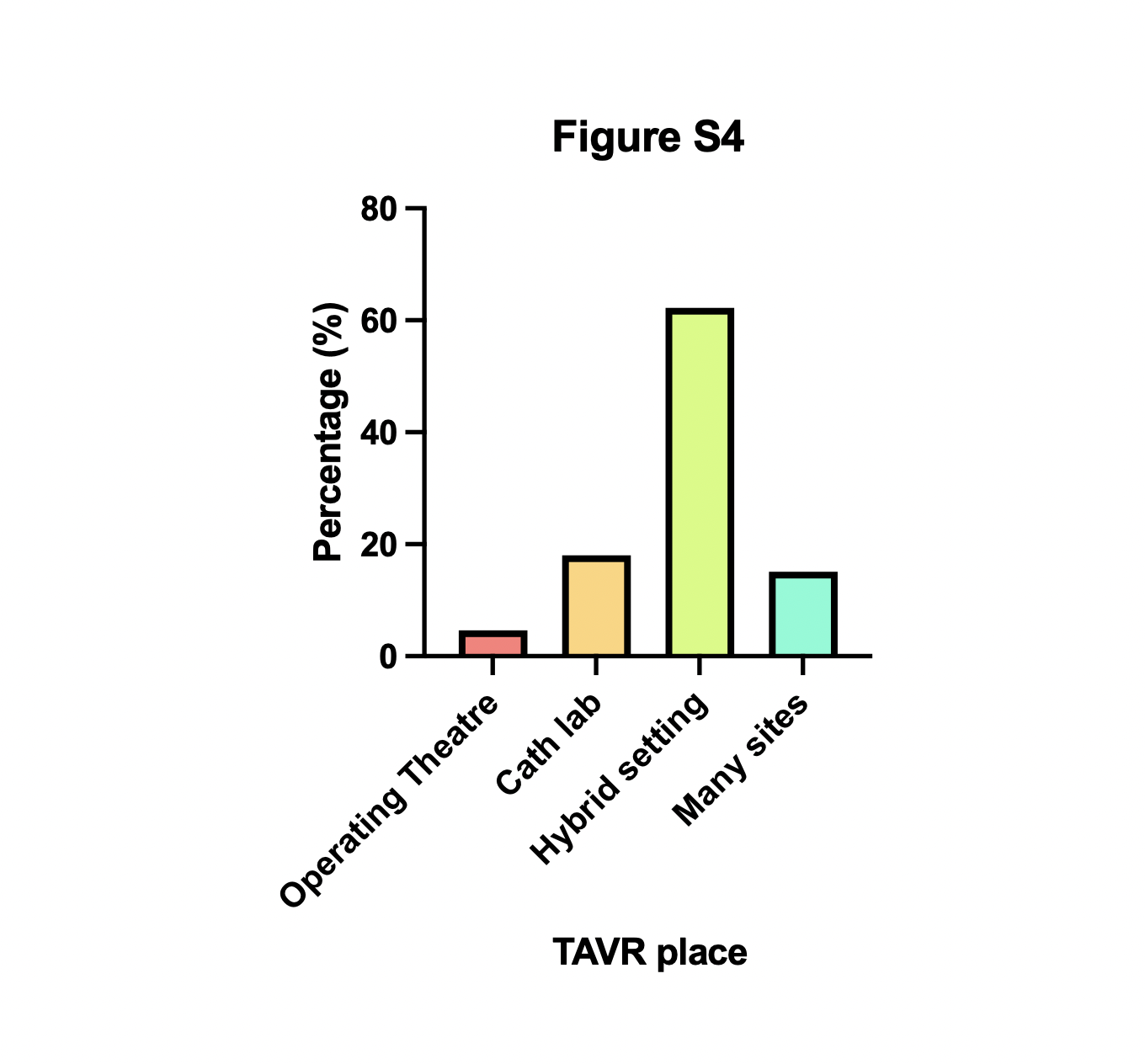
**

**Figure S4:** TAVR place: Operating theatre (salmon), Cath lab (orange), Hybrid setting or room (green), many sites (cyan).

**
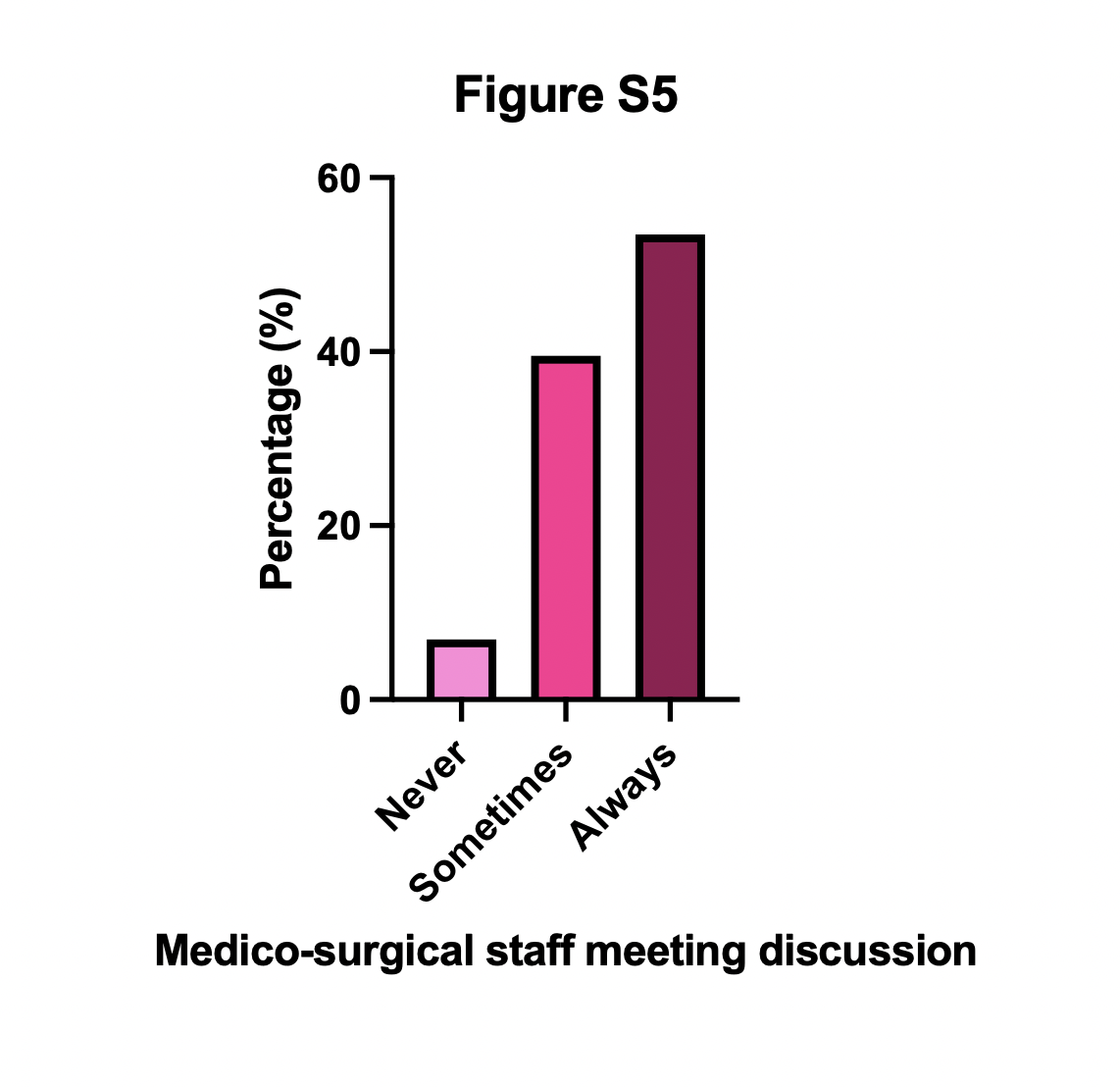
**

**Figure S5:** Percentage of medico-surgical staff discussion, Never (pink), Sometimes (fuchsia), Always (burgundy).

**
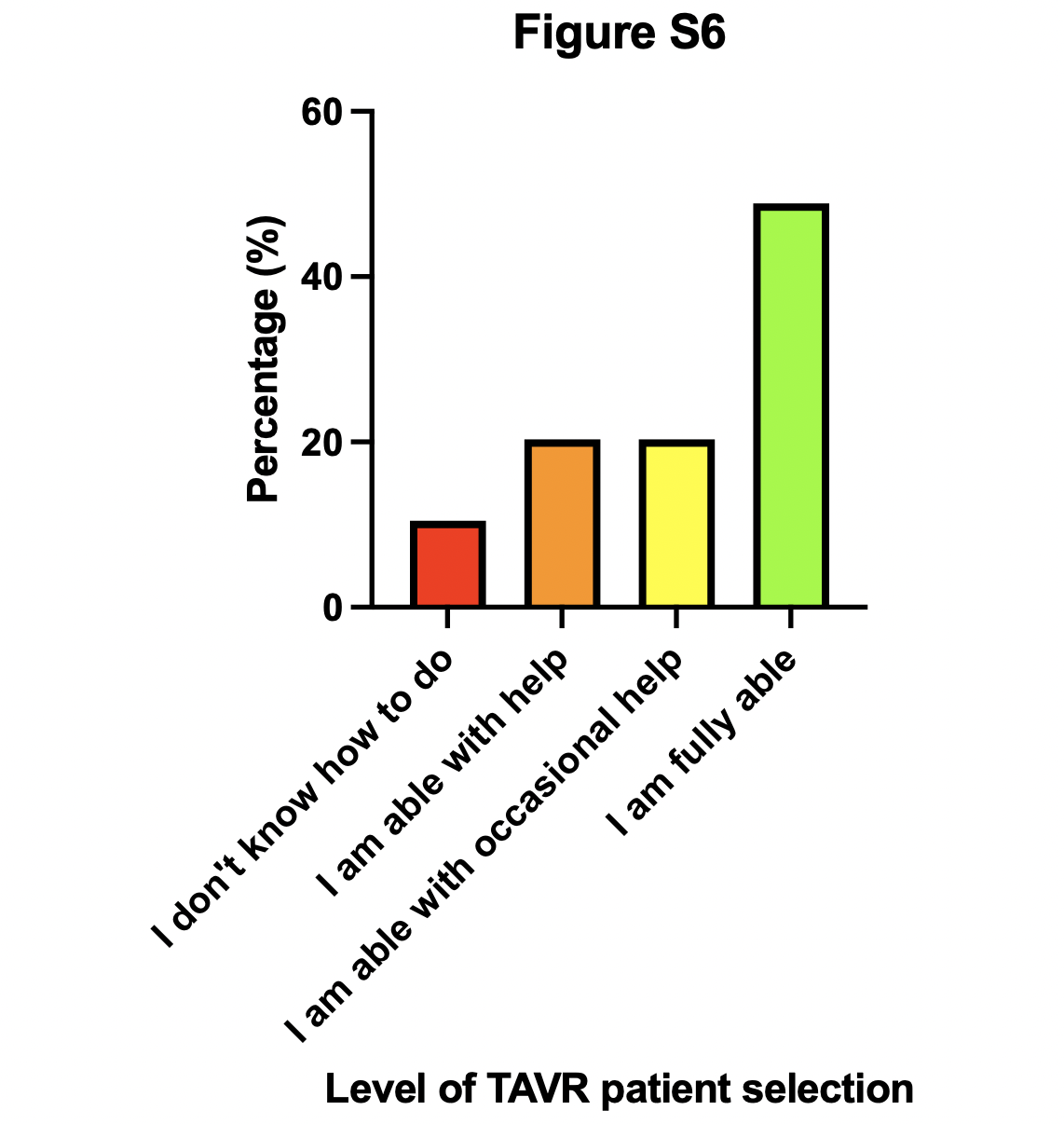
**

**Figure S6:** Level of TAVR patient selection” I don’t know how to do (red), I am able with help (orange), I am able with occasional help (yellow), I am fully able (green).

**
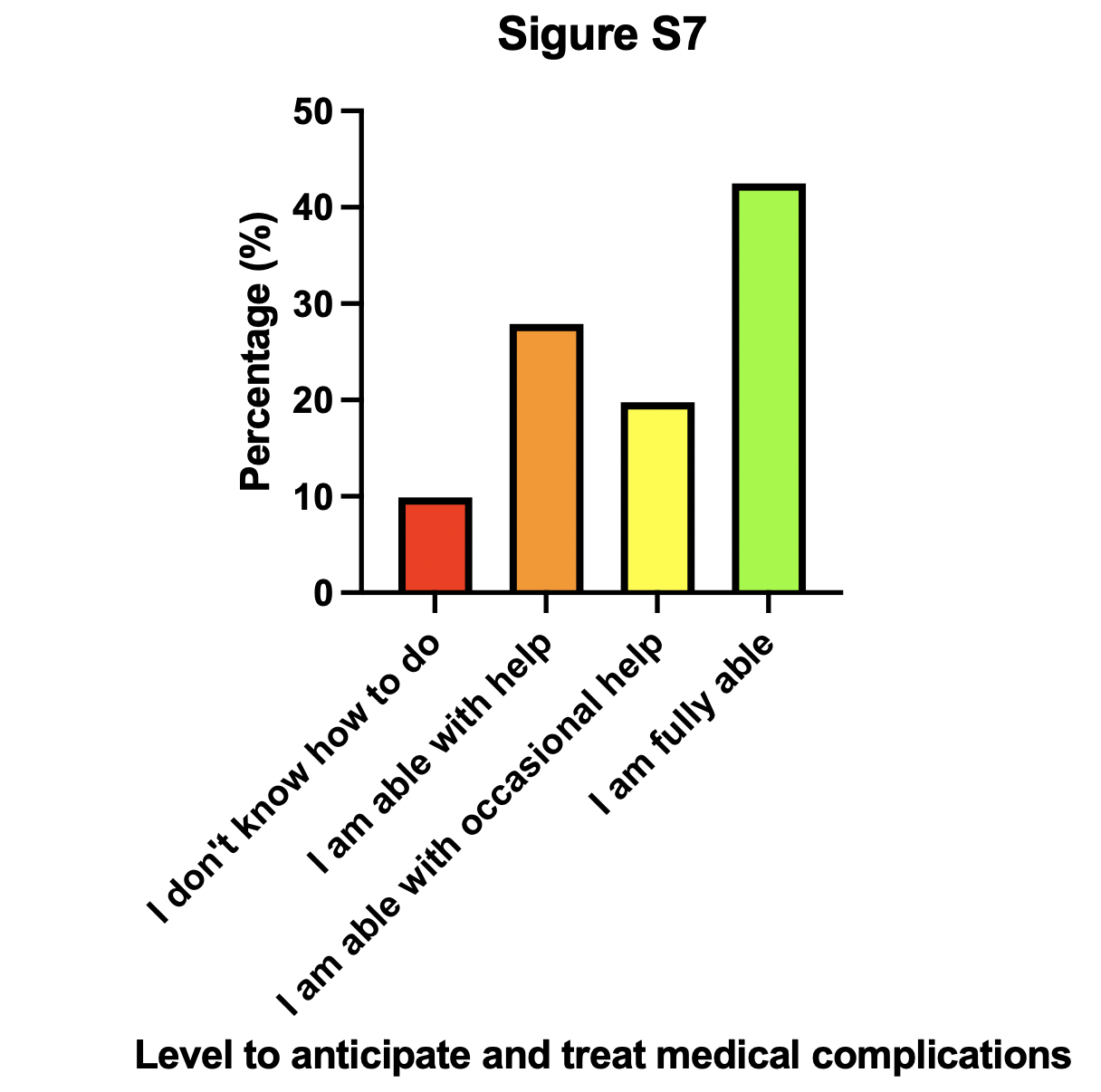
**

**Figure S7:** Level of TAVR anticipate and treat medical complications: I don’t know how to do (red), I am able with help (orange), I am able with occasional help (yellow), I am fully able (green).

**
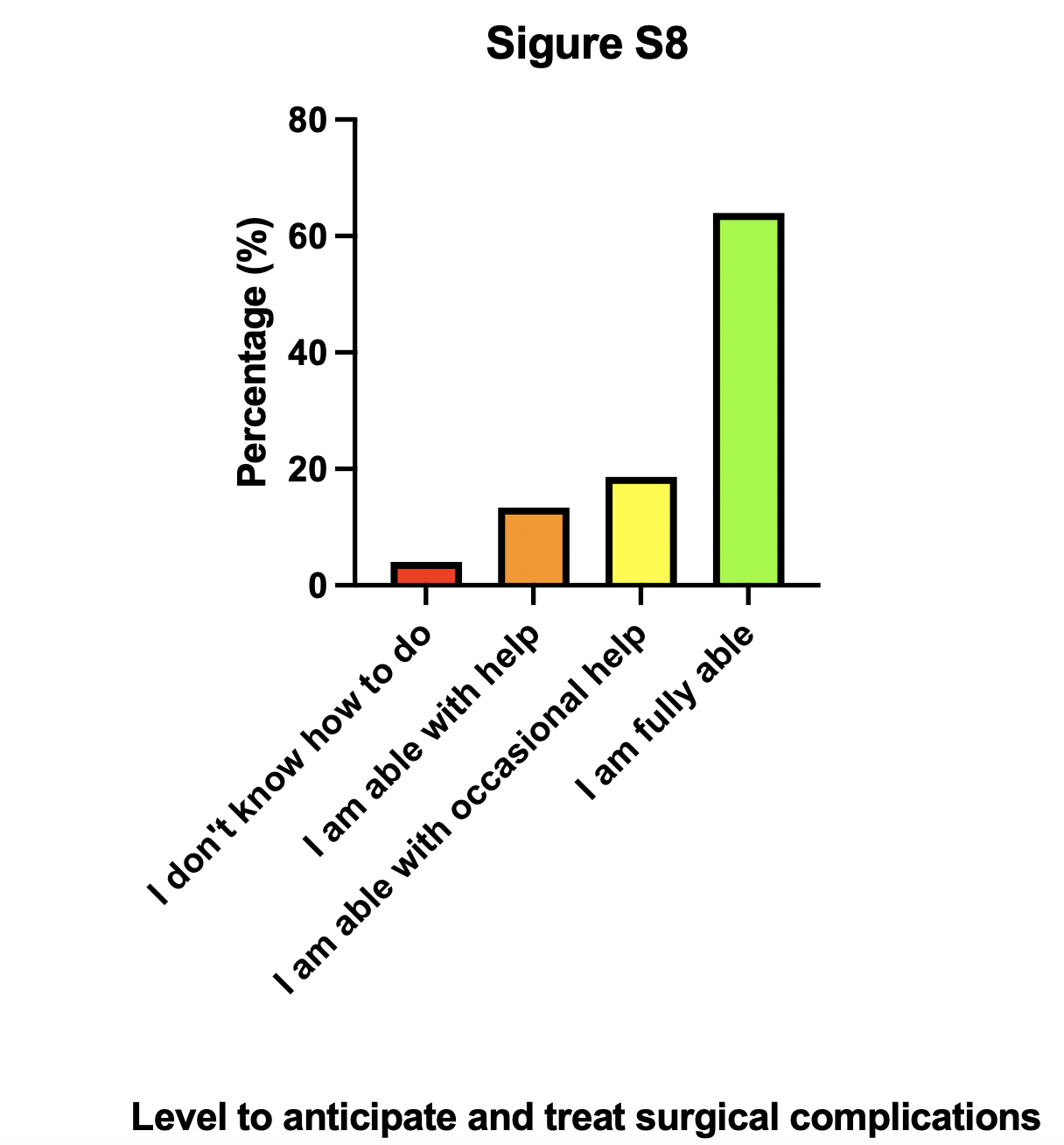
**

**Figure S8:** Level of TAVR anticipate and treat surgical complications: I don’t know how to do (red), I am able with help (orange), I am able with occasional help (yellow), I am fully able (green).

**
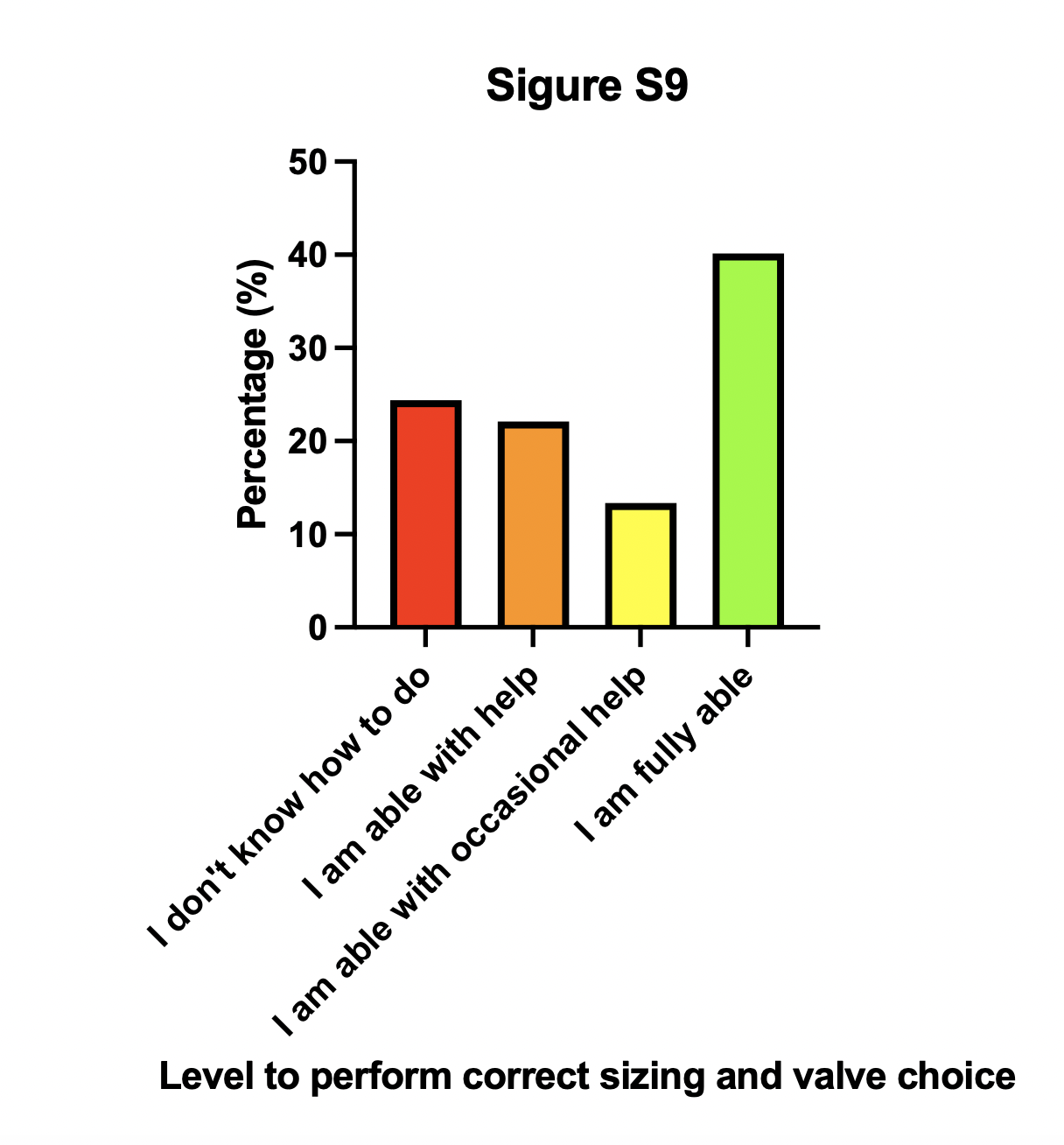
**

**Figure S9:** Level to perform correct sizing and valve choice: I don’t know how to do (red), I am able with help (orange), I am able with occasional help (yellow), I am fully able (green).

**
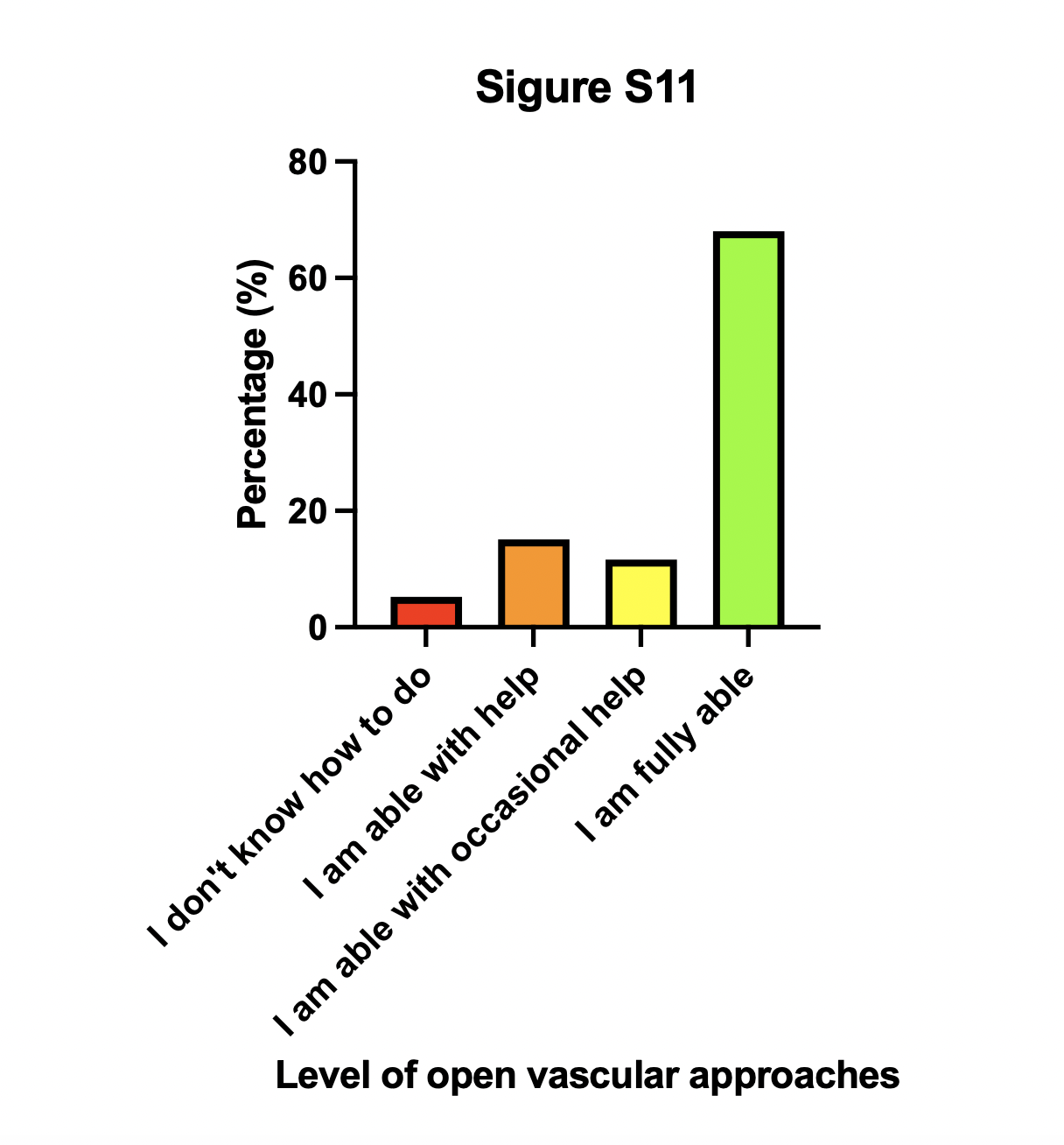
**

**Figure S10:** Level to open vascular approaches: I don’t know how to do (red), I am able with help (orange), I am able with occasional help (yellow), I am fully able (green).

**
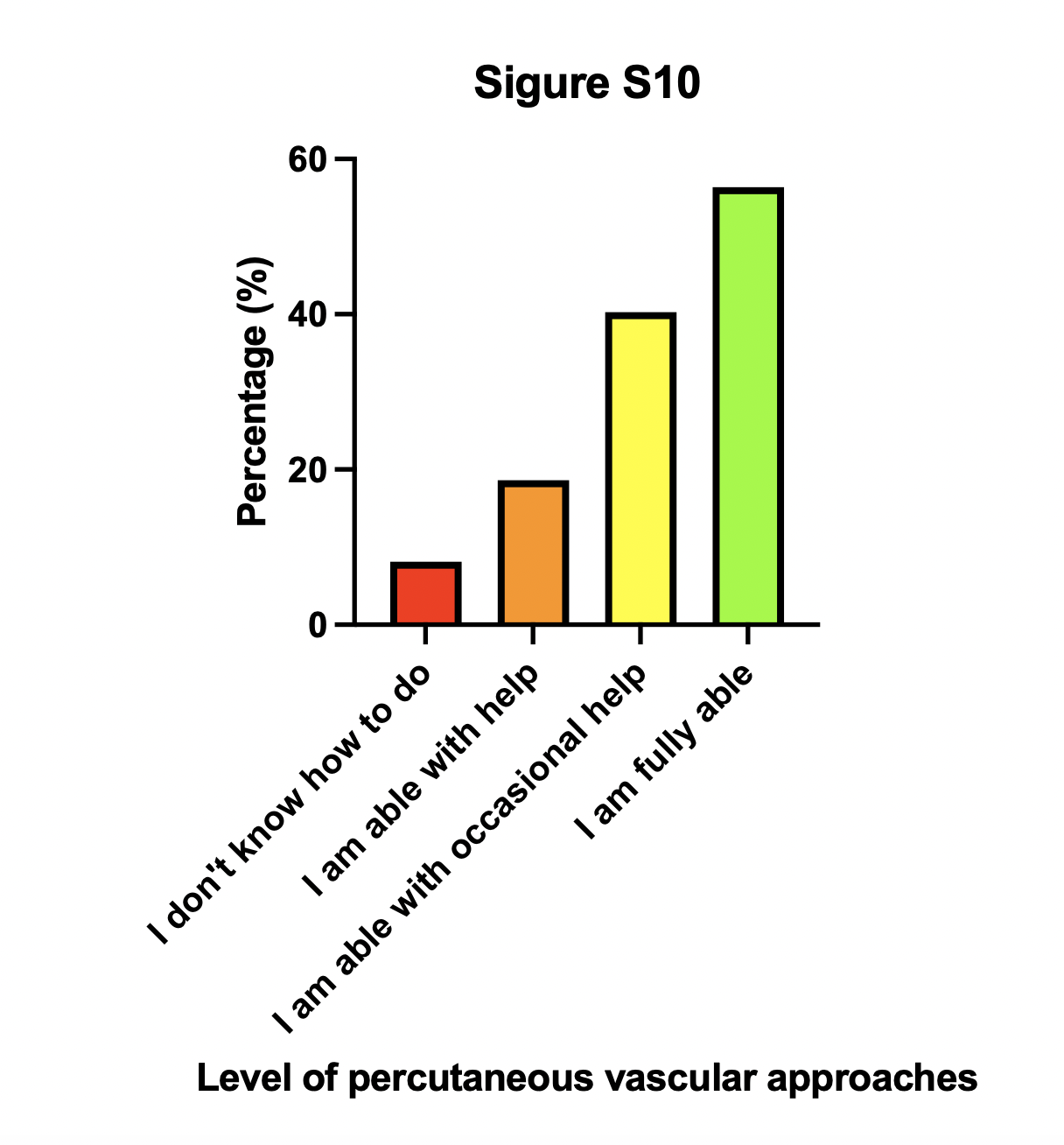
**

**Figure S11:** Level to percutaneous approaches: I don’t know how to do (red), I am able with help (orange), I am able with occasional help (yellow), I am fully able (green).

**
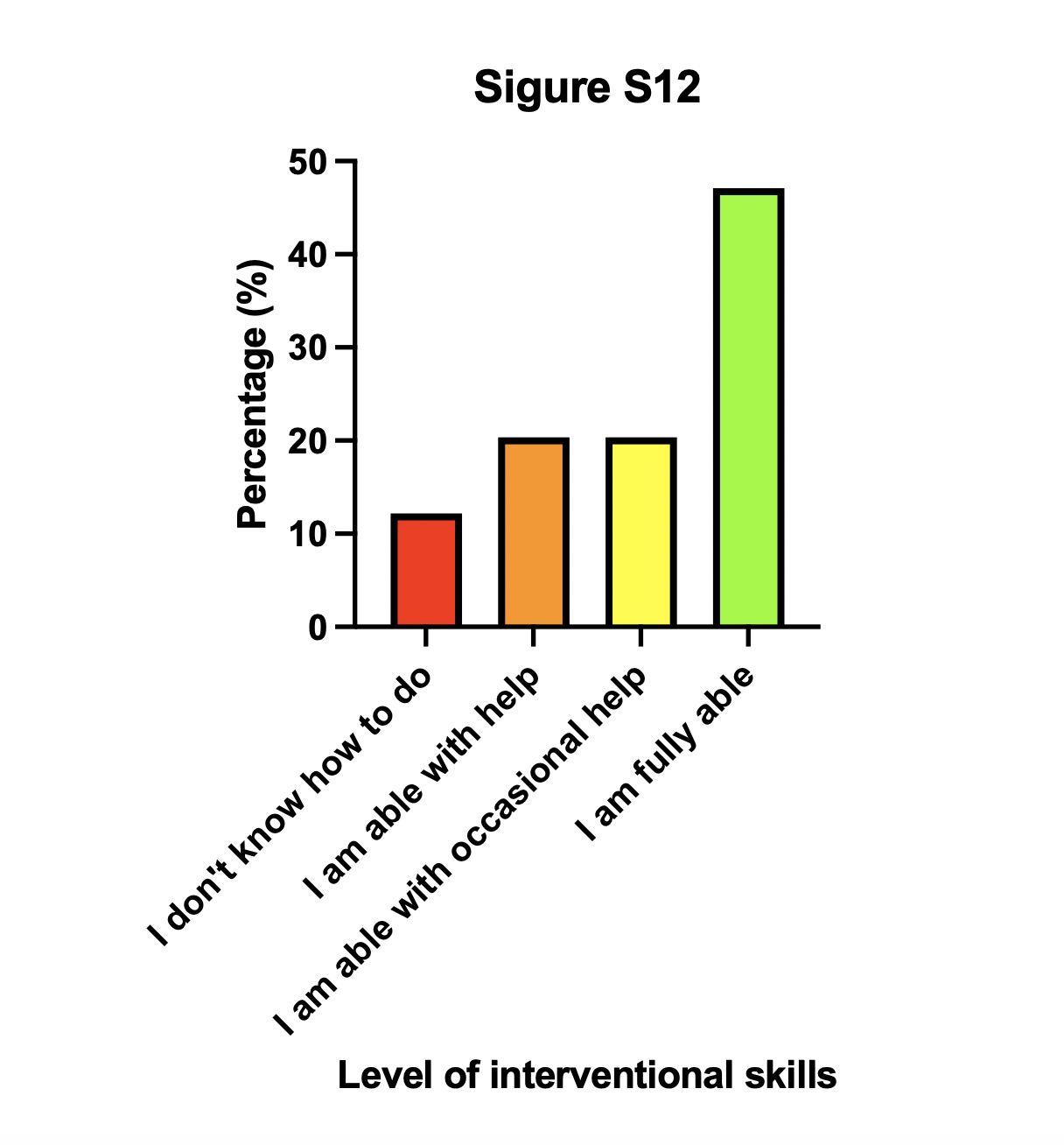
**

**Figure S12:** Level of interventional skills: I don’t know how to do (red), I am able with help (orange), I am able with occasional help (yellow), I am fully able (green).

**
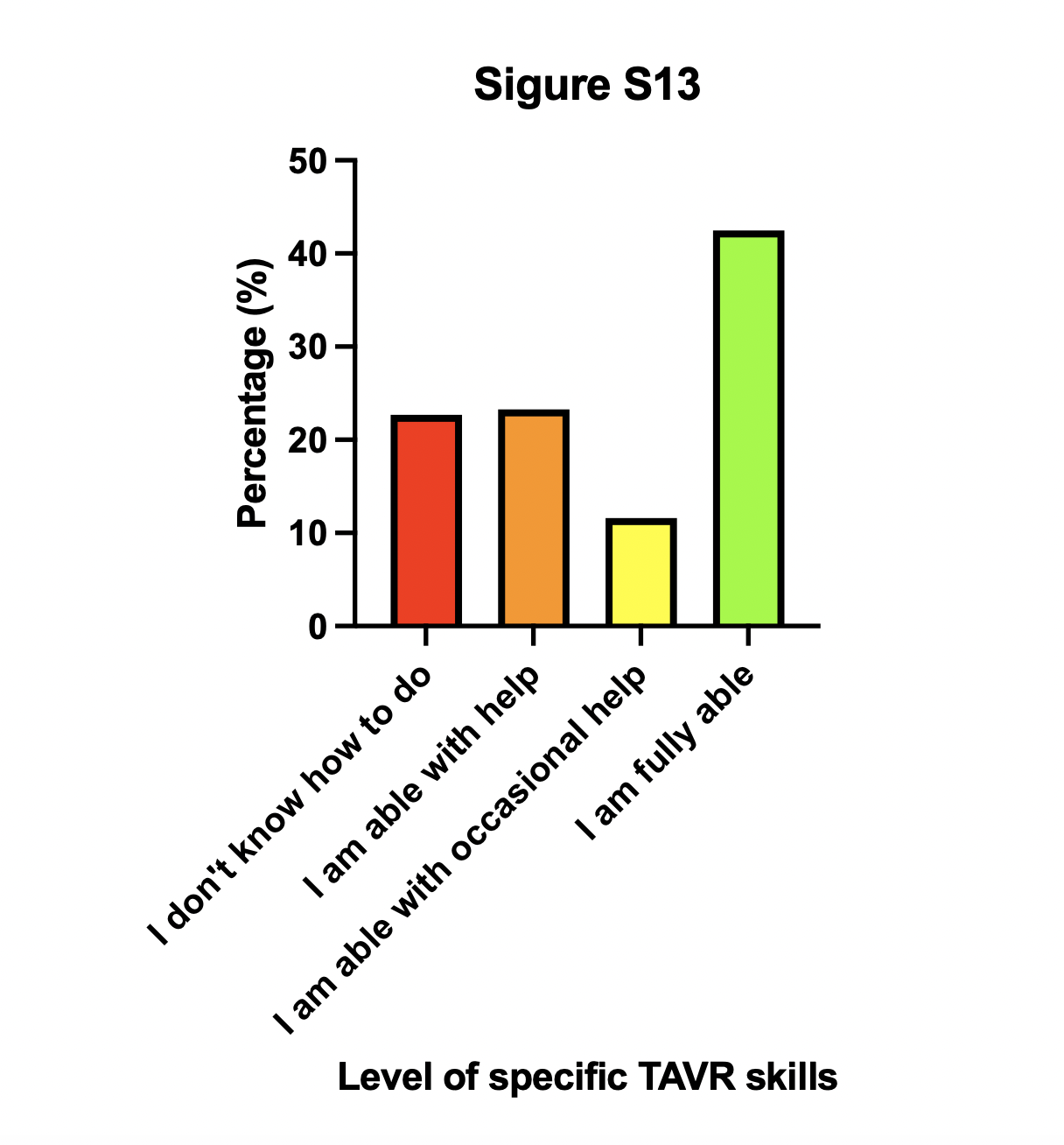
**

**Figure S12:** Level to TAVR specific skills: I don’t know how to do (red), I am able with help (orange), I am able with occasional help (yellow), I am fully able (green).
